# Sharp pain in a normal Achilles tendon of a professional female football player was related to a plantaris tendon in a rare position: a case report

**DOI:** 10.1186/s13256-021-03131-7

**Published:** 2021-10-18

**Authors:** Håkan Alfredson, Lorenzo Masci, Christoph Spang

**Affiliations:** 1 Department of Community Medicine and Rehabilitation, Sports Medicine Unit, Umeå University, 901 87 Umeå, Sweden; 2 Institute of Sports Exercise and Health, University College Hospital London, London, UK; 3 Sports and Exercise Medicine, Queen Mary University of London, London, UK; 4 Department of Integrative Medical Biology, Anatomy Section, Umeå University, Umeå, Sweden; 5 Private Orthopaedic Spine Center Dr. Alfen, Schürerstraße 5, 97080 Würzburg, Germany

**Keywords:** Plantaris tendon, Achilles tendon pain, Tendinopathy, Case report, Plantaris tendon removal

## Abstract

**Background:**

Plantaris tendinopathy and plantaris-associated Achilles tendinopathy can be responsible for chronic pain in the Achilles tendon midportion, often accompanied by medial tenderness. As conservative treatments are less successful for this patient group, proper diagnosis is important for decision making. This report presents a case with plantaris tendinopathy in a rare (superficial) location.

**Case presentation:**

This article describes a pain history and treatment timeline of a professional Swedish female soccer player (32 years old, Northern European ethnicity, white) who suffered from sharp pain in the Achilles tendon midportion and tenderness on the medial and superficial side for about 2 years. Conservative treatments, including eccentric exercises, were not successful and, to some extent, even caused additional irritation in that region. Ultrasound showed a wide and thick plantaris tendon located on the superficial side of the Achilles tendon midportion. The patient was surgically treated with local removal of the plantaris tendon. After surgery there was a relatively quick (4–6 weeks) rehabilitation, with immediate weight bearing, gradual increased loading, and return to running activities after 4 weeks. At follow-up at 8 weeks, the patient was running and had not experienced any further episodes of sharp pain during change of direction or sprinting.

**Conclusions:**

The plantaris tendon should be considered as a possible source of Achilles tendon pain. This case study demonstrates that the plantaris tendon can be found in unexpected (superficial) positions and needs to be carefully visualized during clinical and imaging examinations.

## Background

Chronic pain in the Achilles tendon midportion is a frequent clinical condition among athletes, and especially in elite runners [1]. Treatment in general, but especially during the season, can be challenging [2]. Current scientific evidence recommends slow progressive loading or eccentric training for the majority of athletes [3, 4]. Nonetheless, despite extensive research, there is no consensus on pathogenesis, and the correlation between tendon pathology, pain, and function is not fully understood [2].

Recent studies have identified the plantaris tendon as being involved in a sizeable number of cases with midportion Achilles tendon pain, especially in those cases not responding to conservative treatments [5, 6]. From an evolutionary point of view, the plantaris is a rudimentary muscle, with only minor biomechanical function. In humans, the muscle and insertion sites of the tendon are diverse [7, 8]. Certain anatomical variations can create compressive and shearing forces with the Achilles tendon [9, 10]. This interaction occurs predominately when the plantaris courses close to the medio-ventral aspect of the Achilles or invaginates into the Achilles tendon [5]. This interference is characterized by a co-existing plantaris and Achilles tendinopathy that is clinically characterized by medial Achilles tenderness and localized high blood flow between the plantaris and Achilles [11, 12]. Interestingly in a few of these cases, there is a pathologic plantaris tendon with a normal opposing Achilles tendon, indicating that the plantaris alone can potentially be a driver for Achilles pain [13]. Surgical local removal of the plantaris tendon is often the only definitive option for long-term pain relief [14, 15].

In this case report, we present a rare clinical condition where a pathologic plantaris tendon does not run medially, but superficially to a normal Achilles tendon and causes Achilles pain in a professional female football player. Clinical history, presentation of symptoms, and interventions are described in detail.

## Case presentation

This professional female soccer player (32 years old, Northern European ethnicity, white) reported episodes of sharp pain in the Achilles tendon for about 2 years. During sudden change of direction and sprinting, she described sudden sharp pain in the midportion of the Achilles tendon; a symptom that initially rapidly settled within minutes, and she could continue to train and play. However, over time, her pain episodes increased in frequency and intensity. After a couple of days of rest, her pain would settle and she was able to return to full training. Eventually, she had to cease training and match play. Clinical examination showed a minimal thickening of the Achilles tendon midportion, with some tenderness localized to the medial and superficial side of the Achilles tendon, suggesting a diagnosis of midportion tendinopathy. Although treatment with eccentric training was trialed, these exercises caused more irritation in the affected region. As the condition failed to settle and the athlete could not properly train and play at desired level, she presented for an assessment at our clinic.

**Fig. 1:**
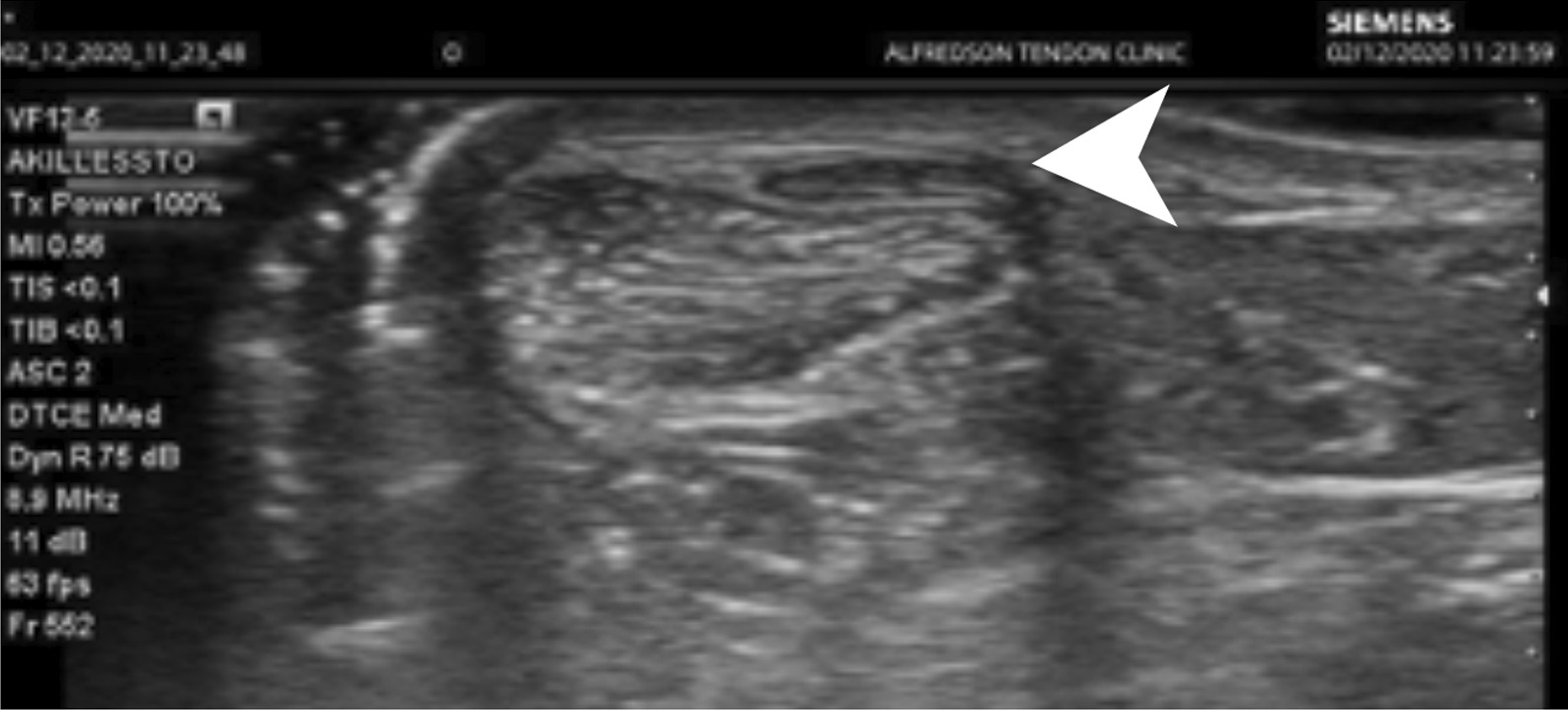
Grayscale ultrasound picture - transversal view showing a normal Achilles midportion and medial on the superficial side of the Achilles there is a plantaris tendon (arrow).

She was a non-smoker, did not abuse alcohol, was on no medication, and had no allergies. During initial clinical examination, blood pressure was 120/70 mmHg and resting pulse 60 bpm. Clinical findings were similar as found previously, with minor thickening in the Achilles midportion and tenderness localized to the medial and superficial side. Ultrasound examination showed a normal Achilles tendon measuring 5 mm in the midportion and with normal blood flow. Dynamic ultrasound scanning identified the plantaris tendon proximally. Following the plantaris distally to the region with localized tenderness, a wide and thick plantaris tendon was located on the superficial side of the Achilles midportion (Fig. 1).

**Fig. 2:**
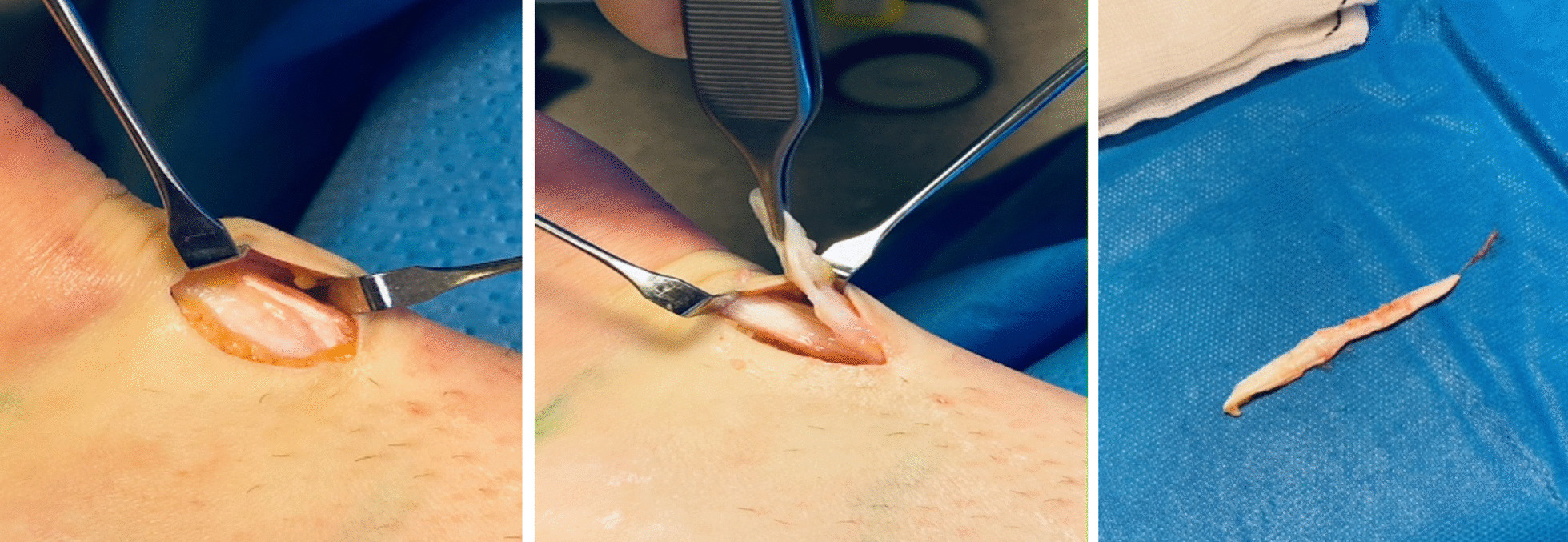
Surgical view-via a short longitudinal incision on the medial side of the Achilles midportion the plantaris tendon is found to be located slightly medial on the superficial side of the Achilles tendon (left). Surgical view showing local removal of the plantaris tendon from the underlying normal Achilles tendon (middle).

The suspected diagnosis was plantaris tendon-related pain, and the patient was surgically treated with local removal of the plantaris tendon performed under local anesthesia (5 ml Xylocaine + adrenaline) (Fig. 2). The procedure took about 30 minutes. During surgery, the thick and wide plantaris tendon was found to be located on the superficial side of the Achilles tendon midportion (Fig. 2). Between the tendons, there was a richly vascularized fatty layer that was scraped loose and removed [16].

After surgery, there was rest with elevated foot overnight, and pain cure using paracetamol 500 mg, 2 tablets every 6 hours during the first 24 hours, if needed. There was a relatively quick (4–6 weeks) rehabilitation, with immediate weight bearing and range of motion (ROM) exercises, gradual increase in loading according to a specific program, and a return to running activities after 4 weeks. At follow-up at 8 weeks, the patient was in full training, and had not experienced any further episodes of sharp pain during change of direction and sprinting.

## Discussion and conclusions

This patient is unique because her Achilles pain was found to be related to the plantaris tendon positioned in an unusual and previously not described position on the superficial side of the Achilles, and not related to Achilles itself. To the best of our knowledge, this has never been reported elsewhere before. Removing the plantaris immediately relieved the pain. The results highlight the importance of proper visualization of the location of the plantaris tendon when examining patients with Achilles tendon pain.

Plantaris tendon-related pain is a relatively new diagnosis, mainly found in a subgroup of elite or recreationally active individuals [5, 7, 17]. There is typically pain on the medial side of a thickened Achilles midportion; sometimes described as sudden and sharp pain, but more often described as dull pain during loading. It has been shown that the plantaris tendon has multiple variable positions in relation to the Achilles [7]. In certain positions, interference between the two tendons is increased, which may explain symptoms variability between patients [9, 10]. Clinical experience suggests that treatment with eccentric training often causes a worsening of pain on the medial side of the Achilles [5]. More commonly, there is a combination of both midportion Achilles tendinopathy and plantaris tendinopathy, but isolated plantaris tendinopathy in close vicinity to a normal Achilles is not unusual [12, 13, 16]. Histological studies have shown sensory and sympathetic nerves in the soft tissues between the two tendons and also within the plantaris tendon [16], signifying multiple potential sites of local nociception. Currently, there is no evidence to suggest that patients with plantaris tendinopathy can improve with conservative treatments alone. From our clinical experience, we feel it is unlikely that high-level athletes can return to previous activity levels without a recurrence of symptoms. Therefore, local surgical removal of the plantaris tendon is often needed. Studies on surgical outcomes have shown positive clinical outcomes, and in patients that also have Achilles tendinopathy improvement in Achilles tendon structure due to the removal of shearing or compressive forces [13–15, 18].

In conclusion, careful visualization of the plantaris tendon is essential for decision making in all patients with midportion Achilles tendon pain, especially in cases with medial Achilles tenderness. This case report demonstrates that the plantaris tendon, while commonly in a ventro-medial position, can also be positioned at the superficial aspect of the Achilles tendon and can cause pain. This further underpins the clinical rationale for a thorough clinical assessment and imaging of cases with Achilles pain, and also shows that the plantaris tendon could be the source of pain.

## Data Availability

The datasets used and/or analyzed during the current study are available from the corresponding author on reasonable request.
